# Can We Identify Non-Stationary Dynamics of Trial-to-Trial Variability?

**DOI:** 10.1371/journal.pone.0095648

**Published:** 2014-04-25

**Authors:** Emili Balaguer-Ballester, Alejandro Tabas-Diaz, Marcin Budka

**Affiliations:** 1 Faculty of Science and Technology, Bournemouth University, United Kingdom; 2 Bernstein Center for Computational Neuroscience, Medical Faculty Mannheim and Heidelberg University, Mannheim, Germany; National Research & Technology Council, Argentina

## Abstract

Identifying sources of the apparent variability in non-stationary scenarios is a fundamental problem in many biological data analysis settings. For instance, neurophysiological responses to the same task often vary from each repetition of the same experiment (trial) to the next. The origin and functional role of this observed variability is one of the fundamental questions in neuroscience. The nature of such trial-to-trial dynamics however remains largely elusive to current data analysis approaches. A range of strategies have been proposed in modalities such as electro-encephalography but gaining a fundamental insight into latent sources of trial-to-trial variability in neural recordings is still a major challenge. In this paper, we present a proof-of-concept study to the analysis of trial-to-trial variability dynamics founded on non-autonomous dynamical systems. At this initial stage, we evaluate the capacity of a simple statistic based on the behaviour of trajectories in classification settings, the trajectory coherence, in order to identify trial-to-trial dynamics. First, we derive the conditions leading to observable changes in datasets generated by a compact dynamical system (the Duffing equation). This canonical system plays the role of a ubiquitous model of non-stationary supervised classification problems. Second, we estimate the coherence of class-trajectories in empirically reconstructed space of system states. We show how this analysis can discern variations attributable to non-autonomous deterministic processes from stochastic fluctuations. The analyses are benchmarked using simulated and two different real datasets which have been shown to exhibit attractor dynamics. As an illustrative example, we focused on the analysis of the rat's frontal cortex ensemble dynamics during a decision-making task. Results suggest that, in line with recent hypotheses, rather than internal noise, it is the deterministic trend which most likely underlies the observed trial-to-trial variability. Thus, the empirical tool developed within this study potentially allows us to infer the source of variability in *in-vivo* neural recordings.

## Introduction

Non-stationary time series are very common in physical and biological systems. Thus, approaches to the analysis of time series in dynamic scenarios have been developed in a wide range of areas such as geophysics (e.g. [1], [2] and references therein), econometrics [3] or human neurophysiology [4] to name just a few. For instance, electroencephalographic responses (EEG) often appear non-stationary; therefore it is crucial to extract invariant, stationary components of the signal for performing reliable analyses [2], [4].

More generally, responses of the brain to the same stimulus typically vary across multiple instances of the same experiment (trials) [5]–[12]. The origin of the trial-to-trial variability is currently one of the most actively debated topics in neuroscience. Trial-to-trial variability has been observed in multiple modalities of neural recordings [5], [7], [13]–[17] and it has been studied using a variety of techniques ranging from multivariate statistics to information-theoretic approaches (e.g. [7], [18]–[20]). However, despite the large number of studies over recent decades, the dynamical substrate of such observed variability is largely unknown [5], [13].

Understanding the main causes of trial variability in neural recordings is a major challenge for current data analysis techniques. Often such variability is attributed to the irregular responses in cortical neurons (due to the probabilistic nature of synaptic transmission; see e.g. [5], [21]–[24]), but other potential causes are the chaotic dynamics of complex neural networks [25]–[27] or the lack of specificity in top-down brain dynamics [13]. Thus it is important to design new data analysis methods in order to discern whether observed variability is essentially driven by stochastic or by deterministic processes.

Data analysis methods for non-stationary environments are a very active research direction in machine learning and computational statistics. Attention has typically been focused on change detection (e.g. [28]–[34]) and on designing strategies yielding to competitive predictions in dynamic settings e.g. in areas such as streaming data mining [29], [35], [36], on-line dimensionality reduction [37], metalearning [38] or Gaussian Processes [39] to name a few. Recent studies identified invariant subspaces, allowing the design of robust models specifically for each stationary data segment [4], [6]. Nevertheless, a common assumption in such approaches is that stationarity is preserved in short segments of the time series (for instance [6]). In this setting, the source of non-stationarity is typically attributed to a “temporal drift” in the statistical moments of likelihood distributions 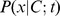, generating 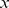 patterns of each class 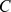 [6], [36].

In this proof-of-concept initial study we propose a different angle for the analysis of multivariate recordings based on non-autonomous dynamical systems. The challenge is to discern whether the observed trial-to-trial variability in recordings is caused by deterministic dynamics or by stochastic fluctuations. Towards this goal, we first analysed a compact low-order nonlinear dynamical system with random initial conditions. As the simplest possible model exhibiting two attractors, we used the Duffing equation[40]–[43], a ubiquitous model arising in many physics and engineering areas such as nonlinear electrical circuits, optics (e.g. [44], [45] and references therein), quantum field theory [45], [46] or the study of chaotic oscillatory behaviour [43]. Similar but less parsimonious multi-stable canonical systems have been recently used for modelling how biological systems effectively operate in non-stationary environments, such as human alpha rhythms underlying EEG recordings [47]. Smooth variations of the high-order perturbation term typically enable such class of models to express a wide dynamic repertoire [47], as is the case in the compact system that we show in this work.

We also propose a simple measure of classifier performance based on the coherent behavior of trajectories with respect to class-boundaries and analyse its response depending on the source of non-stationarity. Time series driven by non-autonomous (time-varying) dynamics show an abrupt variation in the trajectory coherence statistic which is not present in randomly generated data, as commonly assumed in current approaches [29]. Thus, this statistic acts as an immediate signature of a significant variation in the underlying dynamics. Our analyses enable us, for instance, to inform models on the necessity of updating their parameters towards maintaining a competitive performance in non-stationary conditions.

The analysis is then extended to multivariate classification problems in real datasets exhibiting non-stationary dynamics, consisting of atmospheric pollutants and neural recordings time series. As an illustrative example, we focused on multi-unit recordings in rodent frontal cortex networks in behaving animals during the performance of a difficult task [48], [49]. Recently, it has been proposed that behavioural trial-to-trial variability could be the result of the imprecision of top-down processes involved in the performance of cognitively demanding tasks [13], [50], while variability in cell-to-cell responses – the commonly accepted source of the observed variance [21]–[23], [51] – may play a secondary role [13]. Thus, as an illustrative example, we focus on multi-unit recordings in rodent frontal cortex networks. Equipped with the analyses presented here, we suggest that a deterministic trend plays a major role in the observed trial-to-trial variability during decision making.

## Results

The following section introduces intuitively the canonical system used in the study (the Duffing family) and frames it in the context of a supervised machine learning task – classification. This system plays the role of a ubiquitous model for understanding complex classification problems from a nonlinear dynamics angle. Results lead to a proposition in Text S1 and to a general conjecture, which we have benchmarked in real non-stationary datasets discussed in Text S2 and Figure S2. In the last section, these approaches are applied to neural recordings.

### Canonical model of binary classification in non-stationary settings

Our first aim is to infer the conditions in which arbitrarily small perturbations in parameters of underlying dynamics can be discriminated from random fluctuations. The first step is to model a non-stationary two-class classification problem.

The simplest, yet ubiquitous ordinary dynamical system capable of a range of attracting dynamics is the Duffing nonlinear equation, encompassing first order and cubic nonlinearities (the perturbation term) as well as an external force:
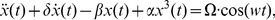
(1)or equivalently,
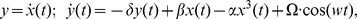
where 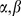 and 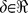 are model parameters. This dissipative autonomous system generates a wide range of attracting phenomena such as bi-stability, periodic orbits and fractal attractors. Thus, it has provided a useful paradigm during recent decades for the study of nonlinear oscillations and chaotic dynamical systems [45]. Despite its simplicity, exact solutions of this system are generally not known, although they have been the focus of many studies during recent decades [41]–[43], [45], thus numerical simulations are needed.

**Figure 1:**
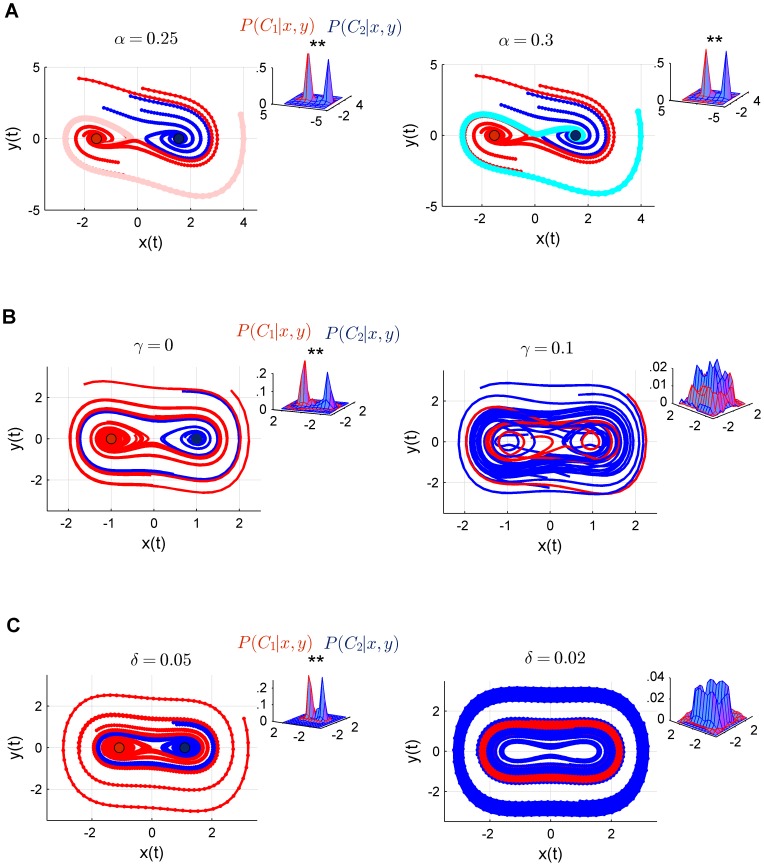
Duffing non-linear oscillator (Equation 1, see parameter values in Methods). (A) A small perturbation leading to a subtle drift in the relative distance between fixed points. Each subplot shows 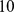 trajectories (i.e. 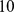 different initial conditions randomly drawn, see text). Light red (left) and blue (right) lines indicate an example of a trajectory that changes its class (i.e. it is attracted to the opposite sink) after the small perturbation induced. Insets show class-posterior probabilities of each phase space vector belonging to the basin of attraction of one of the two sinks (see Methods for details). Two stars (**) indicate significant differences between means in the x-axis at 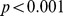; which remain after a subtle variation in the 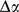 of the perturbation parameter of the Duffing system. (B) and (C): Perturbation in other parameters induces bifurcations leading to chaotic oscillations (B) or global limit cycles (C) e.g. [42]. As in plot A, inset shows the class-posteriors, which are severely transformed after such parameter variations.

For a range of parameter values (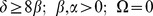) the system has a simple behaviour: a saddle point at 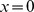 and two sinks at the symmetric equilibrium points 
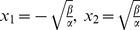
 (Figure 1A; see also Methods).

**Figure 2:**
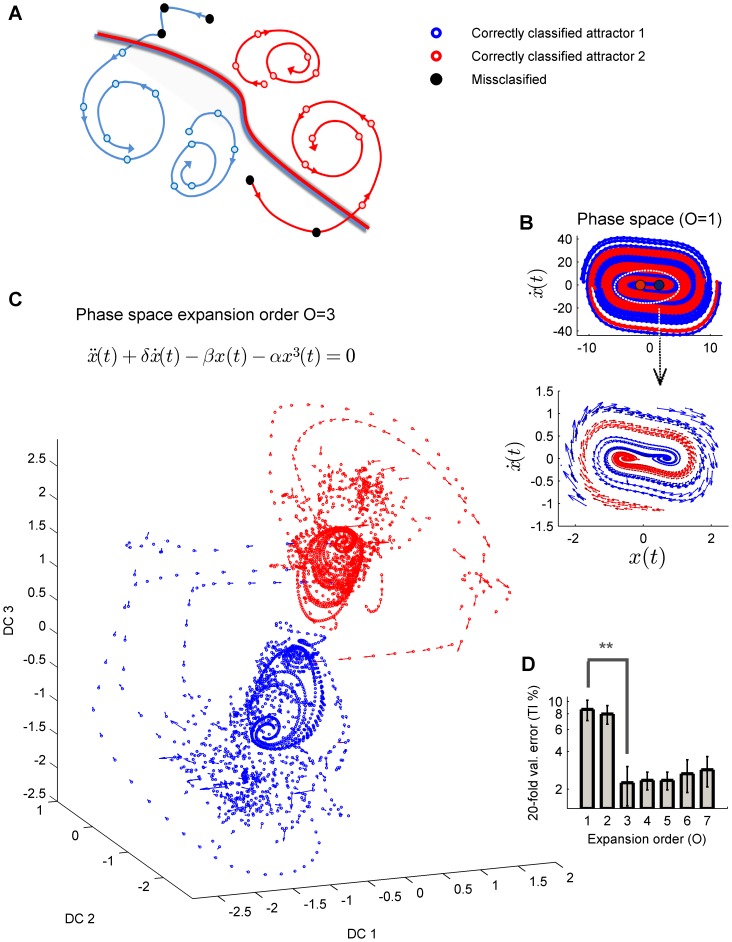
Trajectory behaviour in Duffing systems. (A) Schema illustrating convergent trajectories with respect to attracting state boundaries (see also Figure S1). (B) Phase space flow (using 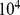 initial conditions). (C) Projection into the three maximally discriminating directions (gram-schmidt ortonomalized) of an expanded space of order three. (D) This optimally regularized discriminant was used to compute the 20-fold cross validation of the trajectory incoherence index (TI) i.e. those different from any of the trajectories shown in plot (A) across initial conditions. The expansion order 3 yields to a maximal out-of-sample convergence; highly significant with respect to the phase space (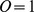) shown in plot B (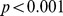, see main text).

A nonlinear two-class classification problem is then naturally defined: Figures 1A and 2B show the basin of attraction of the two sinks, constructed by generating random initial conditions from a fixed, two-dimensional Gaussian distribution centred at the origin (standard dev. 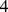), which are then subjected to the flow indicated in Equation 1.

Blue and red dots show fixed points towards which trajectories converge. Trajectories belong to the class 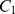 (red) if they are attracted to the left sink or to the class 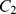 (blue) if they converge to the right sink. Figure 2B shows a more detailed display of the basins of attraction of the sinks (using 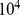 random initial conditions). Groups of class 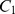 trajectories are interleaved with groups of 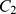 trajectories in the phase space; hence basins of attraction furnish the spiral structure shown in Figure 2B.

Such simple dynamics typically breaks down with changes of 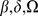 parameters (e.g. it undergoes supercritical pitchfork bifurcation and periodic orbits appear for 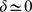, Figure 1B; a chaotic attractor emerges for a range of 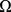 values, Figure 1C [45]), yielding to abrupt variations in posterior probabilities of class-membership 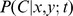 (see insets in the figures and Methods for details).

This setting has parallels with the so-called “concept shift” in data mining literature [38], [52] and is not of interest here as detection of abrupt changes is often successfully addressed by standard change detection approaches (e.g. [29], [36]). Thus, such kind of relatively obvious non-stationary changes, typically induced by bifurcations are not considered in this work.

In contrast, and crucially, here we are only interested in inferring very subtle variations in the underlying system dynamics which are not evident from standard statistical analysis. To this end, we modify slightly the relative distance of the attractors while the dynamics are essentially unchanged by inducing a small perturbation in 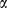, which can be approximated to 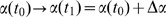 on a first-order level (all other parameters are fixed). As the fixed points become closer to each other i.e. the 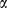 parameter increases (Figure 1A, inset) distribution modes significantly differ (multivariate analysis, Wilks' 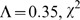 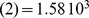, p<0.001). However, some of those trajectories crossing the vicinity of the centre fixed point 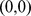 are attracted to the opposite sink i.e. they belong to a different class (Figure 1A). Thus, intuitively, we expect that a classifier which models the two posteriors with negligible error at 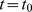, will fail to predict the true class of such trajectories at 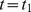 after a subtle drift on the 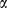 parameter. This arbitrary accurate classifier at 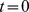 is blind to such a subtle, yet fundamental change in the latent dynamics.

Is there a way to discriminate deterministic variations from changes of probabilistic nature? The following sections show how trajectories which changed the attractor in non-stationary settings allow us to discern the source of the observed data variability.

### Reconstructing attractor dynamics

The analysis starts by devising an optimal classifier for the autonomous (stationary) system shown in Equation 1. The two basins of attractions (the regions of the space in which trajectories ultimately converge towards the corresponding attractor) are not separable in the original phase space (Figure 2B). Thus, an optimally expanded space was used to compute boundaries between classes with a minimum generalization error (the space with the lowest dimensionality allowing us to reach a Bayes-optimal error, see Methods and Figure 2).

Multinomial expansions of a phase spaces are also suitable spaces i.e. the trajectory flow will consistently converge to the corresponding attractor as in the original phase space, while the basin of attraction tends to be linearly separable [48], [53], [54]. Here we used embedding spaces of different dimensionality spanned by high-order interactions up to a 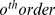 of the original dimensions (see Methods).

In general, distances in such high dimensional spaces cannot be feasibly computed due to a range of problems collectively referred to as the “curse of the dimensionality” in the machine learning literature [55], and especially the distance concentration phenomenon [56]. Thus it is in general not possible to analyse trajectory dynamics directly in large embedding spaces. Nevertheless, a classifier allows us to estimate relative positions of input vectors with respect to the class boundaries (Figure 2). By tracking the predicted label of the 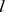 vectors encompassing a single trajectory, we can access and assess the behaviour of the *class*-trajectory in the state space.

In simple terms, a class-trajectory initiated at 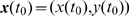 is considered as convergent into a specific volume of the space if *all* its vectors from a certain time 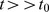 are correctly classified (empirically, it will suffice in this simulation with the last 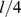 trajectory vectors) i.e. they are assigned to the closer attractor (see schema in Figures 2A and S1A). For instance, trajectories shown in Figure 2A are examples of convergent trajectories, because they either cycle within or finish in the region of the space delimited by its class i.e. its basin of attraction.

We can thus define a natural statistic for time series, the lack of coherence of class-trajectories (trajectory incoherence, TI), as the fraction of complete trajectories which are not convergent. In other words TI is the percentage of trajectories which are *not* of the type of trajectories shown in Figure 2A (see Methods for a more precise definition and Text S1).

TI is thus a quantitative index of trajectory behaviour in non-accessible, high-dimensional state spaces (not to be confused with the exponential divergence of nearby trajectories given by the maximum lyapunov exponent, used as a signature of chaos, for instance [57], [58]). In Figure 2 we estimated TI by cross-validating a regularized Fisher discriminant (*kernelized* for effectively operating in high dimensional state spaces as detailed in Methods [59], [60]). Not surprisingly, an embedding space of third order, precisely the nonlinear order in Equation 1, is the most suitable to capture the attractor dynamics i.e. with the lowest TI. In the light of this simple index, we next studied the behaviour of trajectories in time-varying scenarios.

### Detection of latent non-stationary trends

The analysis continues with a parsimonious simulation of a multi-stage data acquisition setting in noise. We induce a temporal dependency on the perturbation term of the Duffing model (Equation 1),
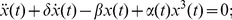
(2)which now has a simple non-autonomous dynamics. We must stress that we are interested here in subtle i.e. non-statistically detectable (on a single-trial basis) variations in the relative position of the attractors in the phase space; which essentially preserve their dynamics (unlike more abrupt non-stationary changes, e.g. Figures 1C and D) therefore bifurcations are typically excluded from this analysis. This subtle non-stationarity is induced by arbitrarily small perturbations in the parameter 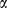, thus, it will suffice to analyse the behavior of TI for a first order expansion of 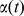 in equation 2. An analysis of the perturbation effect in the system dynamics can be found in Text S1.

**Figure 3:**
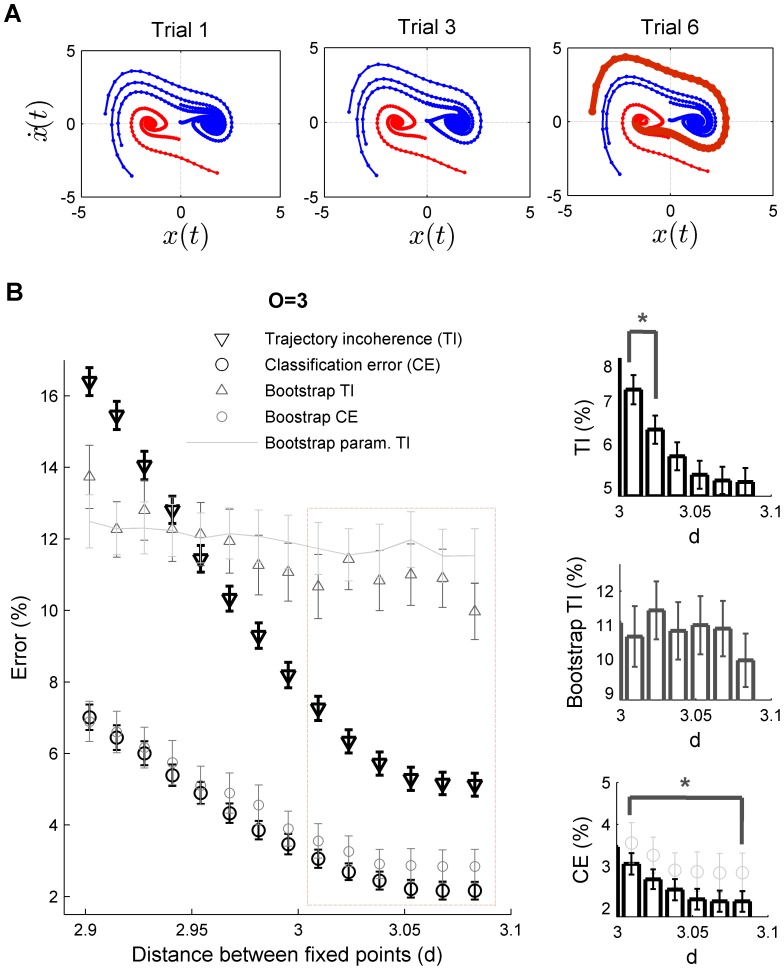
Non-autonomous drift in a non-linear dynamical system (unforced Duffing oscillator). (c.f. Figure S1). (A) Example of a linear variation in the perturbation term 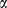 (see also Equation 1). As fixed points approach each other, few trajectories change the basin of attraction and thus the class-membership. (B) Optimally regularized kernel-fisher discriminant in a third order expanded space was used to compute the classification error (CE) and trajectory incoherence (TI) as the distance between fixed point varies (shown mean values of 1000 initial conditions for each trial, error bars are SEM). The discriminant subspace is computed for the first trial and then fixed and applied to subsequent trials (note that only validation results from trials 2–14 are shown in the figure). Insets show amplified versions. Both CE (bottom inset) and TI (top inset) increase over trials, but TI enables us to detect, on a single trial basis, when a significant change occurs. When the temporal contingency within each trajectory is disrupted (bootstrap data, middle inset) TI is no longer sensitive to trial-to-trial variations, indicating the absence of a deterministic trend driving the observed dynamics. When bootstraps are generated by randomly sampling the increment of 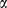 (from a uniform distribution of the same range), no trend in TI is observed either (thin grey line), as expected. These results are fully in line with statistical analyses shown in Figures S1B and S1C.

Figure 3A shows a few randomly generated trajectories, see also schema in Figure S1A. As stated previously, when 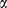 linearly increases the distance between attractors decreases and some trajectories crossing 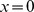 will be potentially attracted to the opposite spiral (see also Text S1). For instance, after six trials in Figure 3A a single trajectory changes the attractor, while no significant change in the statistical moments will be observed, as discussed below.

A simulation of this setting is shown in Figures 3B–D and S1. As expected, the error monotonically increases while distance between fixed point decreases. Critically, there are no statistical differences in the classification error from one trial to the next (two-tailed pairwise t-tests, 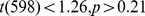, normality accepted according to Lilliefors tests, 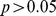). Other standard classification accuracy measures (Wilk's Lambda, higher order statistics such as Jensen-Shanon divergence between posteriors or certainty measures [61]) showed similar insensitivity to those subtle changes (Figures S1B and S2C).

In this simulation, CE does not increase significantly with respect to the first trial before trial number 6 i.e the comparison of trial 1 versus trial 6 is the first to achieve significance (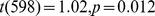, Figure 3B). Thus, when information on the classification performance in previous trials is not accessible, statistics will fail to detect such an event on a single-trial basis. This historical information is often not available.

Class-trajectory coherence statistic (TI), in contrast, allows the detection of such critical change on a trial-by-trial basis. The fraction of misclassified trajectories progressively increases with respect to the previous trial and reaches trial-to-trial significance on trial 6 (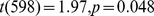) precisely when CE is significant with respect to the reference trial. Therefore TI immediately alerts on the loss of generalization capability of the classification model, unlike the classification error and related statistics (Figure 3C, thick triangle markers). Consistently, the Priestley-Subba-Rao test (PSR) of non-stationarity shown in Figure S1C (see Methods, [32]–[34]) is non-significant for all trial-to-trial pairwise comparisons of 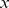 and 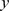 time series until trial 5 (non-parametric MannWhitney 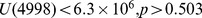); while it reaches trial-to-trial significance precisely on trial 6 (Figure S1C, MannWhitney 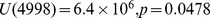; normality rejected according to Lilliefors test, 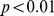) fully in line with TI results.

Note also that initial conditions were randomly drawn from a normal distribution spanning up to four standard deviations, suggesting that TI is robust to high levels of this input noise at 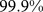 confidence. However, and significantly, this is only the case if the underlying source of non-stationarity is deterministic. Figure 3B also shows bootstrap data, constructed by shuffling vectors 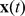 within each trajectory, while class-associations are maintained. Thus, CE is not altered, but the temporal flow within trajectories breaks down. In this setting, there is no guarantee that trajectories are attracted to any volume and thus TI should not vary significantly (Figure 3B, grey triangle markers), suggesting that multi-stable deterministic dynamics does not play a major role in the observed data. Likewise no trend in TI is observed either when trajectories are preserved, but the perturbation term varies randomly from trial to trial; in other words when the autonomous duffing system is deterministic but its non-autonomous dynamics is stochastic (grey line in Figure 3B), as envisaged.

These results have been illustrated for the Duffing family, but this analysis potentially has a wider scope of application.

As a simple, intuitive example, consider an autonomous (static) dynamical system parameterized by 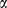 equipped with i.i.d. random initial conditions; this system generates an observable dataset of 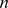 trajectories of length 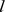 patterns each. Consider also an accurate classifier in a Bayes sense for such stationary dataset. Then, a small parameter perturbation such as the ones illustrated in Figures 1 and 3 will have a completely different effect on CE and TI. Since at least one trajectory of length 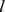 will converge to a different attractor (see also Text S1 Lemma 1), the change on TI is at least 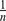:
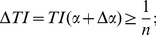
(3)


By definition of TI only the last 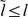 vectors from a trajectory of length 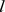 will be misclassified, thus:
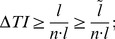
(4)


As the classification error is the fraction of misclassified vectors 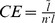, trivially, the following relation holds:
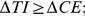
(5)


This is precisely the result shown in Figure 3C i.e. TI increases more abruptly than the classification error.

In contrast, if we consider an identical dataset in which *all* 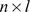 patterns (not just the initial conditions) have been i.i.d randomly drawn i.e. where there are no coherent trajectories, a change in the parameters of the generative distribution does not guarantee Equation 5 bound. Thus, TI would not be sensitive to any changes and CE would be a more appropriate estimate in this i.i.d. data. This effect is shown in Figure 3C, where the order or vectors within trajectories has been randomly altered before the system undergoes a parameter drift (Bootstrap TI in Figure 3C).

The approach devised here could be thus applied to multi-stable scenarios, where a “snapshot” of attracting dynamics is observed in each trial. As a real data example, we applied analyses in a well-known, multivariate time series where attractors subtly drift over time, discussed in detail in Text S2. The dataset consists of hourly concentrations of ozone, meteorological variables and other atmospheric pollutants (Text S2). Ozone time series are well known-to exhibit daily periodicity which is modulated by a subtle seasonal trend [62], [63]; thus they will serve to benchmark further simulation results before the analysis of neural data in the next section.

This first illustrative analysis is shown in Figure S2. Precisely as in the dynamical system simulations, a signature of non-autonomous dynamics is indicated by an abrupt increase in TI not accompanied by a sudden change in CE, suggesting a deterministic trend in the observed trial-to-trial variability (see details in Figure S2 and Text S2).

In summary, results obtained for the Duffing family of dynamical systems are potentially extendible to more general settings, exhibiting a repertoire of attracting dynamics in noise. The next section shows another example of application of our approach, the investigation of trial-to-trial variability in *in vivo* recordings.

### Trial to trial variability in neural ensembles

Neuronal responses to the same task often differ from trial to trial, particularly when recorded in higher cognitive areas [5]. The origin and functional role of this variability has recently attracted a lot of attention in neuroscience [5], [13], [64], and has been analysed using a variety of statistical and information-theoretic approaches (e.g. [6], [7], [18]–[20], [65]).

**Figure 4:**
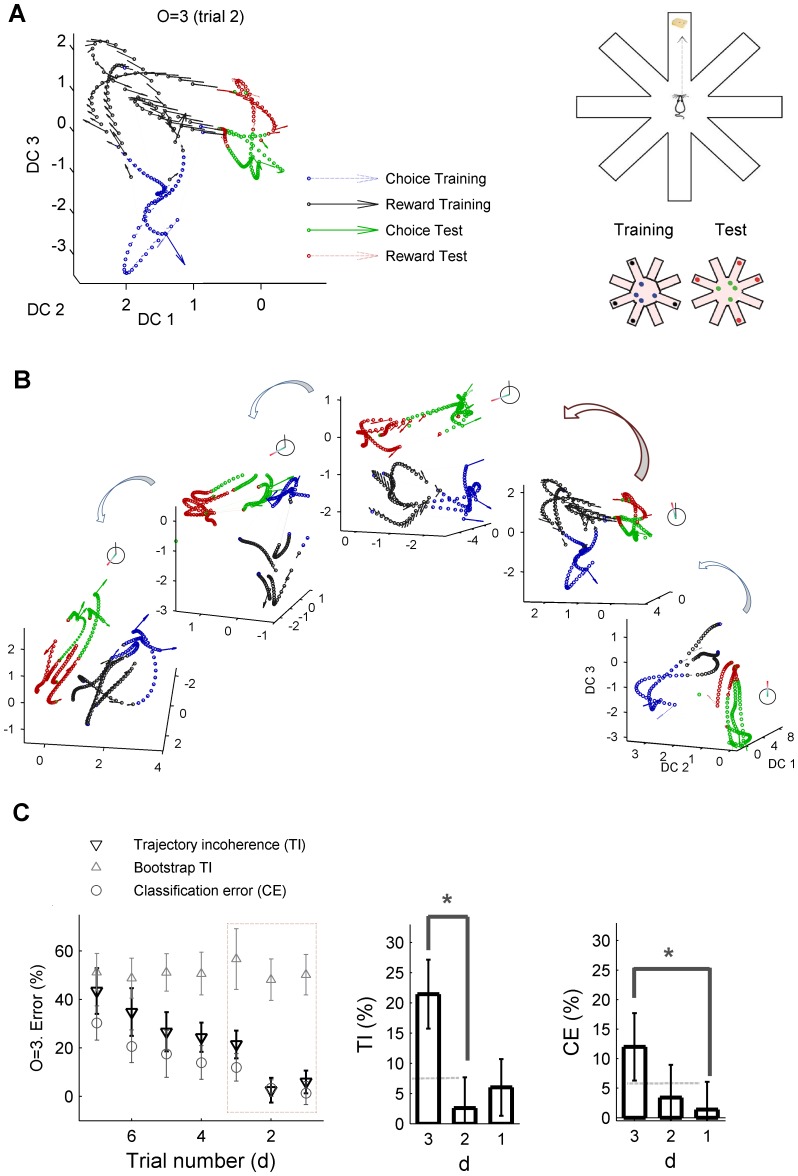
In vivo neural ensemble recordings in rat frontal cortex. (A) Example of a delay-coordinate map expanded to a third order state space; see Methods and [48]) projected onto the three maximally discriminating dimensions (ortonormalized). Different colours correspond to different stages of the task (a radial arm-maze, inset left). (B) A clockwise rotation of the task-stage states from trial to trial seems to take place, suggesting a deterministic drift in the putatively attracting sets associated with task epochs. (C) Non-stationary drift in ensemble recordings. Analyses on an expanded space of third order where optimised for the first trial, the maximally discriminant subspace is fixed and then used to compute CE and TI in the next trials. As in the theoretical model (Figures 1–3) and in the real data example (Figure S2), TI increases faster than CE. Again consistently with previous results, when temporal order of vectors is shuffled, TI is not sensitive to trial-to-trial shifts in dynamics.

The analysis developed in this work enable us to infer whether the observed trial-to-trial variability is essentially driven by stochastic processes as typically assumed in previous studies. We focus on a cognitively demanding task to investigate the trial-to-trial dynamics of neural ensemble recordings in rodent frontal cortex. Figure 4A shows an example of a memory-guided decision- making radial arm-maze experiment (e.g. [48], [49]). In a nutshell, the animal visits a series of baited arms during the training phase (termed choice epochs) in order to consume the reward (termed reward epochs), followed by a delay phase in which no task is performed (omitted in the Figure). Subsequently, during the test phase, the rat visits different arms to obtain the reward again. Activity of a neural ensemble was recorded in a rat frontal cortex during several consecutive trials (Methods). We next defined a classification problem where classes correspond to short (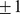 sec.) temporal periods surrounding choices and reward epochs during training and test periods, respectively (the rest of the firing rate vectors are not considered in the analysis). For more details on the task, see Methods and [49]). Figure 4B shows the projection into the three maximally discriminating dimensions of the optimally expanded space. In this case the reconstruction started with a delay-coordinate map before the nonlinear expansion map [53], [54], [66] as a previous step for disambiguating the trajectory flow (see [48], [67]). As in Figure 2, arrows indicate the flow field of neural population states; which moves quickly between different task phases, suggesting the presence of attracting states. Attracting dynamics of neural ensembles have previously been found in different areas such as the olfactory bulb of insects, rodent hippocampus [68]–[71] and in prefrontal cortex [48].

However, Figure 4C also shows responses from trial to trial subtly differ: there is an apparent clockwise rotation of the task-epoch trajectories suggesting a consistent temporal drift, which may be the cause of such non-stationarity. The approach developed here helps to discern whether the origin of such shift can be solely attributed to stochastic fluctuations.

A sufficient condition of non-autonomous dynamics is a sharp increase in TI index just at the trial when the classification error significantly increases (with respect to any previous trial); as devised in the previous section. This is precisely the result of the analysis shown in Figure 4C, where TI abruptly changes on the third trial (Mann-Whitney 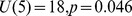; normality rejected according to Lilliefors test, 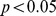). As in Figure 3, this trial-to trial variation is non-significant for CE by large margins (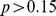 for any trial-to-trial test comparison) while the comparison of trials 1 and 3 CE reaches significance (Mann-Whitney-U, 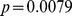)

In order to ensure further the significance of these analyses, bootstraps were constructed by shuffling the firing rate vectors within trajectories while preserving the trials order [48]. According to previous section results, 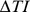 should no longer be informative, as shown in Figure 3C. This prediction is again fully in line with results reported in Figure 4C.

Overall, Figure 4 shows that during the performance of this cognitively demanding task, the process underlying trial-to-trial variability in frontal cortex ensemble recordings is essentially non-autonomous. The aim of this single example is only to illustrate the capacity of the proposed approach. However, this striking result suggests that intrinsic, random fluctuations may not be the only cause of the observed variability in ensemble recordings, as commonly assumed in neural modelling [5].

## Discussion

In this proof-of-concept study we devised a sufficient condition to identify when a multivariate dataset has undergone changes in its parameters' dynamics from trial to trial. The proposed statistic, class-trajectory coherence (or lack thereof) is an easily accessible value, sensitive to subtle departures of deterministic nature in multi-attracting dynamics subject to input noise. This analysis is particularly advantageous when statistical moments do not significantly vary from trial to trial and thus a significant trend cannot be statistically proven on a trial-to-trial basis by standard testing.

The fraction of non-coherent trajectories is a sufficient statistics i.e. if data is independently drawn, both trajectory and classification errors would behave similarly, indicating that deterministic hypothesis cannot be accepted. The importance of this study hence also stems from the fact that i.i.d. data generation is still the typical assumption in current data mining approaches for non-stationary problems [36]. For i.i.d. data, the classification error or derived measures are appropriate empirical estimators of the “true error” (the asymptotic risk, a well-known result in statistical learning [72]) and trajectory analyses are not necessary. A number of tests for non-stationary time series have been proposed in the statistical literature based e.g. on fourier analyses [33], [34] or more recently on wavelet spectrum analyses (for instance see [32]); such tests are also powerful tools when the sampling size is significant (unlike the *in vivo* ensemble recordings analysed here).

As an example of a real-world application, we used two well-known and completely different datasets where attracting dynamics was observed. The main focus of our analysis was on in-vivo neural ensemble recordings, where trial-to-trial variability is often observed. The origin of trial-to-trial variability in neural recordings is a fundamental question in neuroscience, touching the essentials of our understanding of neural computations. Among the many possible causes, it has been traditionally accepted that the intrinsic irregularity of spike probability is the origin of most of the observed trial-to-trial variance, mainly due to probabilistic nature of synaptic transmission [5]. Thus, very recently, efforts have been applied to devising suitable methods for the analyses of non-stationary spike trains[6], [65]. In a similar spirit, recent models have sought to infer time-varying statistics of synaptic conductances from membrane recordings (e.g. [73]–[75]).

However, there are no empirical demonstrations of whether internal, random fluctuations always drive the observed trial-to-trial variance in neural recordings. The hypothesis stating that the observed trial-to-trial variably has a stochastic, internal origin has recently been debated [5]. For instance, Beck and colleagues [13] proposed that spike irregularity is often a minor contributor to the unexplained variance, while suboptimal inference (the imprecision associated with deterministic approximations in complex computations) may be the dominant component of behavioural variability in difficult tasks. Thus, most of the variability may be originated rather by complex or chaotic deterministic processes [13], whose parameters can be top-down modulated by active attention (e.g. [50], [76]) or by stimulus expectancy [18].

The analyses performed within this study are in line with this hypothesis: we have observed that trial-to-trial variability processing in frontal cortex has a deterministic component. Nevertheless, in this work we show only a limited dataset as an illustrative example because our focus here is rather methodological (an exhaustive analysis on ensemble recordings is not in the scope of this preliminary study).

Our initial analyses are also potentially relevant in the context of biophysical modelling. It has recently been proposed that structured stochastic fluctuations have a highly beneficial function by enhancing the dynamical repertoire of multi-attractor landscape of deterministic networks shaped by anatomical structures in cortex [15], [64]. In contrast, in other contemporary models, the richness of observed activity pattern dynamics is provided by purely deterministic, transient dynamical objects. Such *heteroclinic channels* [77], [78] are not attractor states, but still retain the neural activity trajectories only for a limited amount of time, even without the intervention of stochastic variability. The class-trajectory coherence statistic presented here would help to validate empirically or disconfirm these two theories.

In a wider scope, understanding the dynamics underlying non-stationary recordings is a ubiquitous problem of computational biology and data analysis. Contemporary machine learning approaches focus on designing algorithms capable of operating in non-stationary settings (e.g. [30], [36], [37], [52]). In this context, the results of this study suggest that trajectory coherence can be used to detect when a classifier needs updating on a single trial basis. This is a critical advantage of our method as with sufficiently smooth drift, an arbitrarily large number of historical results may otherwise be required, which is often computationally impractical in real life settings (e.g. in data streams or online settings [30]) and sometimes not even experimentally accessible.

In summary, in this opening work, we have provided simulated and real challenging scenarios where standard statistics are unable to identify a deterministic trend on a trial-by-trial basis. Analyses developed in this study help to circumvent drawbacks of existing data analysis tools in order to potentially enable a deeper insight into the dynamic sources of the observed trial-to-trial variability in neural recordings.

## Materials and Methods

### Analyses

#### Compact non-autonomous dynamical system

The unforced Duffing oscillator for 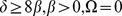, as indicated in the Results section, has a simple behaviour consisting of three fixed points (two spiral sinks and a centre). Trivially, the linearized system matrix,
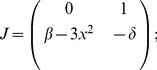
(6)has eigenvalues 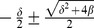 for 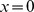 and 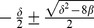 for the two attractors 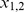 (e.g. [42]). The basic set of parameters used in static simulations (Figures 1–2) were 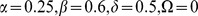 (Figure 1A, left plot, Figure 2B) [42]. In Figure 1, only the parameter specified in the plot title is varied, while the rest of parameters are held constant.

A discrete trajectory of class 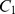 (c.f. 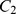) of length 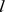 is defined as
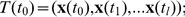
(7)where 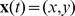, the initial condition 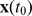 belongs to the basin of attraction of the positive attractor (blue, class 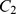; c.f. red, class 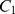) i.e. the continuous counterpart of such discrete trajectory asymptotically converges to the two fixed points 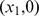 (c.f. 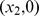.

In Figures 1 and 2, a class 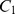-trajectory is a set of 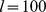 consecutive patterns with a random initial condition 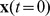 i.i.d. drawn from 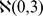 such that 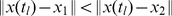 (c.f. 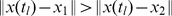 for class 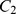).

Posterior probability distributions shown in Figure 1 (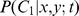 and 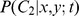) are computed by tiling the phase space in equal rectangular bins; the limits of the grid are defined by the maximum and minimum values of x and y axes in each simulation. The histogram of classes (i.e. of the corresponding attractors of phase space vectors) is then computed and normalized, yielding to posteriors estimates.

The model used in this work is the simplest dynamical system that can implement a binary classification problem (as defined herein). Although exact solutions are generally unknown, approximations can be established (e.g. Text S1, [45]) enabling us further insights into the system dynamics. A more detailed study of the behaviour of the non-autonomous Duffing oscillator can be found in Text S1.

#### Reconstruction of attractor dynamics

Kernel algorithms (e.g. [31], [59], [79]) were used to solve the non-autonomous classification problem in a phase space where basins of attractions are separable. Recently, embedding delay-coordinate maps were combined with nonlinear expanded spaces to reconstruct neural activity trajectories [48]. A polynomial expansion of a phase space is a potentially valid reconstruction of attractor dynamics in moderate noise conditions (for instance [53]) and a well-know reproducing-kernel Hilbert space [59]. Thus, an expanded space of dimension 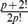 is devised here by including high-order interactions up to a 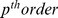 of the phase space variables. The dot product of two feature vectors is the inhomogeneous polynomial kernel of a Mercer type [59], [80],
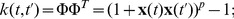
(8)


A regularised kernel Fisher discriminant was then 20-fold cross-validated (Figure 2C, D) in blocks of 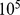 patterns (1,000 trajectories of 100 patterns each on this test set). Optimal regularization penalties, specific of each expanded space, were previously established on an independent (validation) dataset leading to the minimum TI index; see details of this process in [48], [60]. Normality is preserved in the discriminant subspace (Lilliefords non-parametric test, 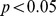) as expected from the Central Limit Theorem [55], [59], [81], leading to a negligible cross validation error for the optimal expanded space (see Figure 2D).

Figure 2A shows an intuitive schema on the class-trajectory coherence index (TI). To be more precise, consider an autonomous dynamical system parameterized by 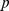 coefficients 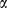 in a dynamical regime corresponding to multiple attracting sets:
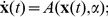
(9)where 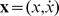 is a 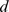-dimensional phase space and 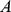 is a nonlinear differential operator.

This system, equipped with i.i.d. initial random conditions, defines a natural classification problem. The system generates an observable dataset 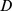 of size 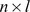 patterns (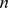 discrete trajectories of length 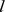). In this context, 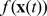 is an arbitrary classifier such that the “true” (asymptotic) risk [6], [59], [72] 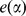 given that the pattern **x** belongs to class 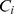


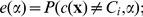
(10)is minimum. The empirical estimator of the true error is the classification error CE shown in the figures. Taking into account the definition of class-trajectory (Equation 7), we term 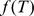 as the predicted class for each point in the trajectory
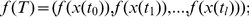
(11)


Thus, a divergent or incoherent class-trajectory is the one in which *all* vectors from a certain 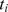 are incorrectly classified. In other words, considering trajectory of class 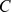 i.e. in which all points of the trajectory belong to this class, a divergent class-trajectory verifies
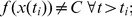
(12)


For simplicity, we will indicate the last condition as 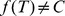. The true trajectory error is then
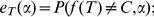
(13)


The lack of trajectory coherence index, TI, shown in figures is the empirical estimator of the true trajectory error 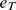.

#### Analysis of the non-autonomous system

Endowed with the definition of TI, we can infer the conditions for a classifier to be no longer optimal when the system undergoes gradual non-stationary drift. In short, Text S1 analyses show how an arbitrarily small parameter perturbation 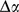 causes at least one trajectory to change its basin of attraction i.e. its class as was demonstrated empirically in Figures 1–3. In Figure 3 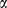 increases by 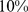 after each time step. The dataset size is the same as in the previous sections (1000 randomly generated initial conditions i.i.d. normally drawn, zero mean and 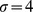).

As suggested in this section, 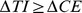 cannot be established in general: for i.i.d. data from a generative distribution 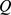, the change induced in the distribution parameters 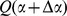 does not necessarily entail a change in TI. For instance, given 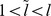 misclassified i.i.d. patterns, the log-likelihood that they belong to the same trajectory is typically very small, and thus we cannot expect a different behaviour of TI and CE statistics (Figure 3C, TI bootstrap; see also Figures 4 and S2 bootstrap data).

The classical Priestley and Subba Rao (PSR) test of non-stationarity (Figure S1C) was used to analyse the simulated dataset shown in Figure 3, because it typically requires large sample sizes for a robust estimation (e.g. [32], [34]). The simplest version of the test consists of analysing the logarithmic of the time-varying spectrum,
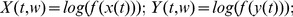
(14)where 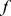 is an estimator of the fourier spectrum and 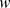 is the frequency. The logarithm typically stabilizes the variance and thus enables us to assume a linear model for 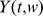, 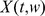 with constant covariance. Differences between non-stationary means in segments of 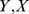 are then analysed using standard statistical testing [32], [34] as shown in Results section and in Figure S1C.

### Data acquisition

#### Behavioural task and electrophysiological recordings. Electrophysiology and preprocessing

The animal recorded was treated in accordance with the ethical guidelines set forth by the Canadian Council for Animal Care. Procedures have been approved by the Animal Care and Biosafety Committee of the University of British Columbia (UBC) and conform to the UBC policy 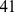 regarding research and teaching involving animals. For a detailed description of the surgical and probe making procedures see [48], [49]. In brief, electrophysiological data was recorded via a 24 single-wire tungsten array implanted into the ACC of the behaving rodent; recordings were sampled at 30 kHz, band-pass filtered from 600–6000 Hz. Spike channels were then amplified, sorted and classified offline using the Spikesort 3D unsupervised clustering software (Neuralyx; Bozeman, MT, USA) as explained in [49].

Spike trains from the 24 simultaneously recorded units were convolved with Gaussian functions to obtain statistically reliable estimates of spike densities. The value of the optimal bandwidth for each neuron (variance of the gaussian kernel) was optimized using a multivariate kernel density estimation approach as described in [82] (see also [83]). Spike density estimates were then binned at 100 ms, so that 95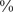 of bins contained 1-0 spikes.

#### Behaviour

Behavioural data were captured via a video camera (Cohu, Poway, CA), recorded in Noldus Ethovision (Noldus, Leesburg, VA) and also stored for off-line analysis. The rat was trained on the delayed spatial win shift run on an eight arm radial arm maze where all arms where initially baited. Each trial consisted of a training, test phase (separated by one minute delay not considered in this study). During the training phase, four of eight arms where opened to enable acquisition of a sugar reward (Noyes, Lancaster, NH). After the delay, all eight arms were opened during the test phase and errors were scored as re-entries into previously visited arms (Figure 4A). This task was performed ten times (trials). The animal scored no error during this task in any of the trials.

In this study we focused on four periods with different cognitive demands, namely reward epochs (dark gray and red dots) during the training or test phases, respectively and correct choice epochs during training and test phases (blue and green, respectively). Reward epochs were defined as the 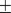
1 s periods around the point in which the animals nose reached the sugar pellet; similarly choice epochs were defined as 1 s periods around each arm entry (see [48]).

#### Standard statistical testing, atmospheric pollution supplemental dataset and software

Statistical test details can be found in the corresponding sections. Nonparametric tests were used based on conservatively designed bootstrap data (200 replications used for two-sided comparisons at 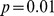, [81]) as explained in the corresponding text sections and figure captions.

Analyses presented in this work are also benchmarked with an additional illustrative dataset where the presence of attracting states is well-known. Data used in this research belongs to the Department of Agriculture, Generalitat Valenciana (Regional Government), Valencia, Spain; and it was recorded in a rural area of particular agricultural interest. Data consists of hourly concentrations of ozone, 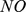, 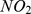 and hourly recordings of meteorological variables for over a two month period. Ozone concentration is known to exhibit regular daily oscillations yet subtle seasonal variations [62], [63]and thus this data is an ideal testbed for the TI index. Details of this dataset and analyses performed can be found in Text S2 and Figure S2.

Software for analysing trajectory dynamics is freely available under the terms of the GNU licence as Software S1. Updates of this software are available at http://www.bccn-heidelberg-mannheim.de and http://www.researchgate.net/profile/EmiliBalaguer-Ballester/ websites.

## Supporting Information

Figure S1Non-autonomous drift in the duffing dynamical system (cont. from Figure 3). (A) Schema illustrating convergent trajectories with respect to attracting state boundaries in the reference set (top left), in the prediction (validation) set after a deterministic drift preserving the initial conditions (top right) and when those initial conditions are randomly drawn (bottom); the later setting is related to the analyses shown in Figure 3. As illustrated in the figure, the behavior of CE and TI indexes is remarkably different. (B) The left axis shows the Jensen-Shannon divergence between predicted posteriors provided by the discriminant analysis (same dataset as in Figure 3). As in Figure 3 analyses, regularized kernel-fisher discriminant in a third order expanded space was optimized for the first trial and applied to the subsequent trials. As the distance between fixed point varies, like in CE, the Jensen-Shannon divergence increases approximately monotonically in a logarithmic shape, thus it is not sensitive to any change in dynamics (two-tailed t-tests, 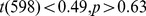, normality accepted at 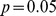 according to Lilliefors test). The right axes show the Wilks 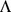 value, which behaves in similar way to CE and Jensen-Shannon divergences. All trial-to-trial comparisons are again non-significant (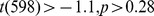, normality accepted at 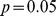). Moreover, the first significant result is achieved in the pairwise comparison form trial 1 to trial 6 (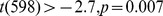), fully in line with CE results shown in Figure 3. (C) Priestley-Subba-Rao test (PSR) of non-stationarity [32]–[34](see main text and Methods). Again fully in line with TI results (Figure 3) only the pairwise comparison from trial 5 to trial 6 reaches significance (MannWhitney 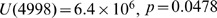; normality rejected according to Lilliefors test, 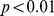).(TIF)

Figure S2Example of the analysis of a non-stationary dataset. (A) Hourly ozone (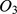) ground concentration, nitric oxides (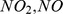) temperature and relative humidity during a summer week. Ozone is an atmospheric pollutant synthesised primarily from 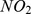 (red line in the plot) by the catalysis of solar radiation. Ozone levels are divided into three ranges (low, moderate and high). (B) An optimally regularized discriminant defined in an expanded phase space of third order is used to map precursors and atmospheric variables to 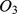 classes. As in Figure 3, the discriminant subspace is computed for the first trial (i.e. the first week of data) and then used to compute CE and TI on the next trials. In week 6, an abrupt increase of TI is not accompanied by a trial-to trial change in CE, suggesting a deterministic origin of the observed non-stationary in hourly ozone concentrations. Lowest plot shows the certainty in the classification (see Text S2).(TIF)

Software S1 Demo trajectories reconstruction toolbox; pls revise this cite in the text and EM.
(ZIP)

Text S1 Local trajectory analyses in a Duffing system.
(PDF)

Text S2 Illustrative dataset in a non-stationary environment.
(PDF)
