# Supplementary figures and images for: Can We Identify Non-Stationary Dynamics of Trial-to-Trial Variability?
Source: PLoS One. 2014 Apr 25;9(4):e95648. doi: 10.1371/journal.pone.0095648 (PMC4000201; doi:10.1371/journal.pone.0095648)

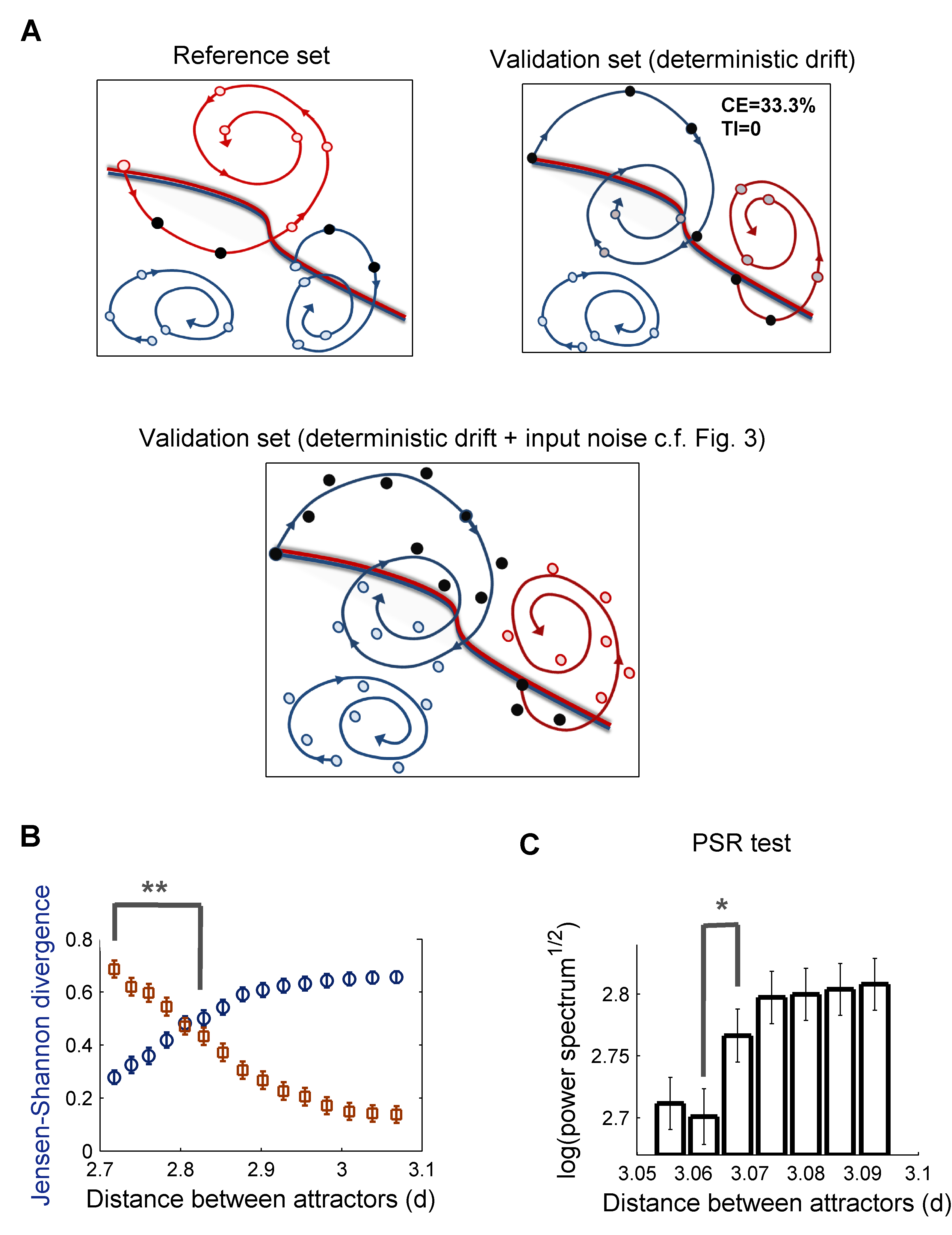

Supplement: Figure S1 — Non-autonomous drift in the duffing dynamical system (cont. from Figure 3). (A) Schema illustrating convergent trajectories with respect to attracting state boundaries in the reference set (top left), in the prediction (validation) set after a deterministic drift preserving the initial conditions (top right) and when those initial conditions are randomly drawn (bottom); the later setting is related to the analyses shown in Figure 3. As illustrated in the figure, the behavior of CE and TI indexes is remarkably different. (B) The left axis shows the Jensen-Shannon divergence between predicted posteriors provided by the discriminant analysis (same dataset as in Figure 3). As in Figure 3 analyses, regularized kernel-fisher discriminant in a third order expanded space was optimized for the first trial and applied to the subsequent trials. As the distance between fixed point varies, like in CE, the Jensen-Shannon divergence increases approximately monotonically in a logarithmic shape, thus it is not sensitive to any change in dynamics (two-tailed t-tests, , normality accepted at according to Lilliefors test). The right axes show the Wilks value, which behaves in similar way to CE and Jensen-Shannon divergences. All trial-to-trial comparisons are again non-significant (, normality accepted at ). Moreover, the first significant result is achieved in the pairwise comparison form trial 1 to trial 6 (), fully in line with CE results shown in Figure 3. (C) Priestley-Subba-Rao test (PSR) of non-stationarity [32]–[34](see main text and Methods). Again fully in line with TI results (Figure 3) only the pairwise comparison from trial 5 to trial 6 reaches significance (MannWhitney ; normality rejected according to Lilliefors test, ). (TIF) [file pone.0095648.s001.tif]

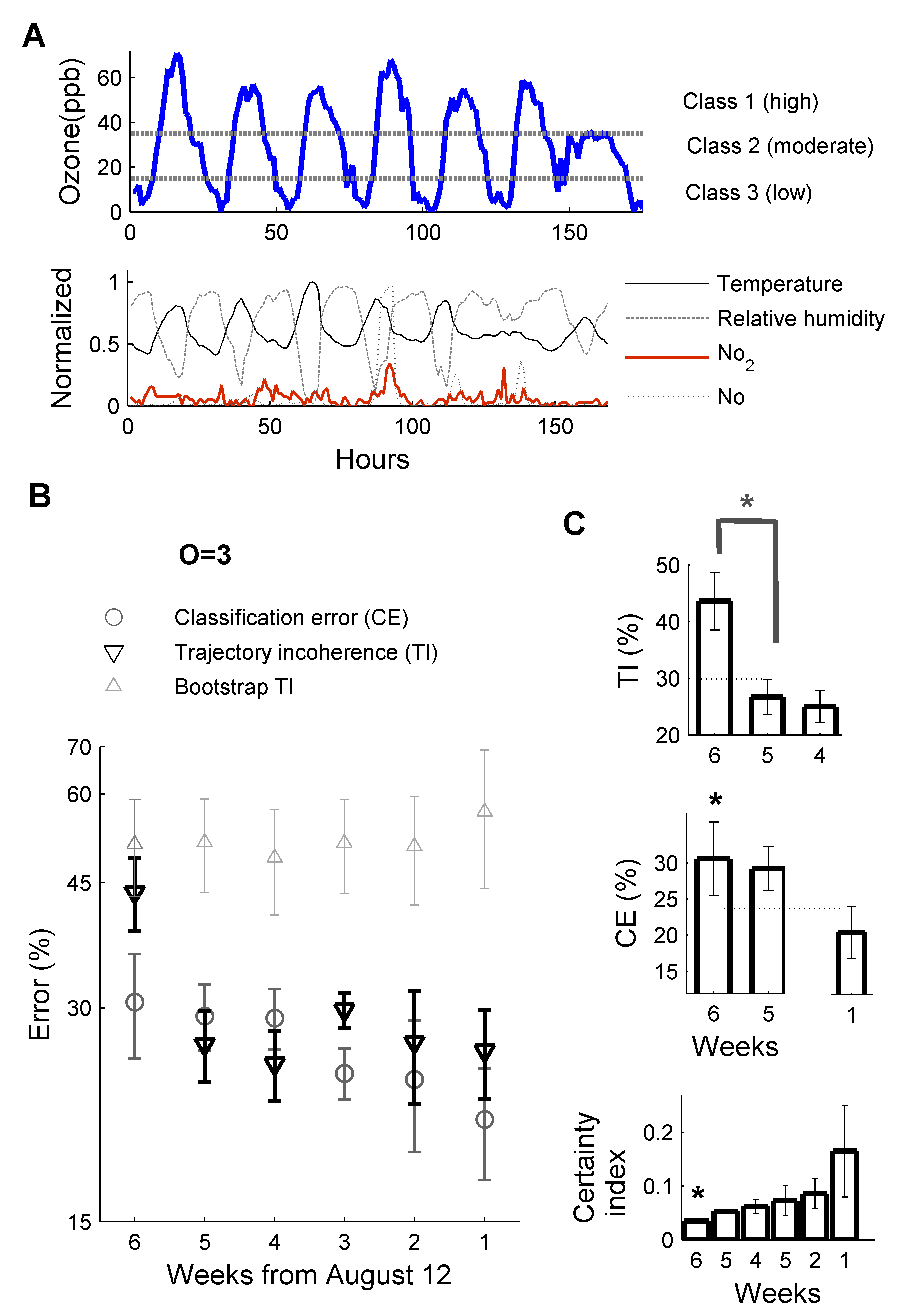

Supplement: Figure S2 — Example of the analysis of a non-stationary dataset. (A) Hourly ozone () ground concentration, nitric oxides () temperature and relative humidity during a summer week. Ozone is an atmospheric pollutant synthesised primarily from (red line in the plot) by the catalysis of solar radiation. Ozone levels are divided into three ranges (low, moderate and high). (B) An optimally regularized discriminant defined in an expanded phase space of third order is used to map precursors and atmospheric variables to classes. As in Figure 3, the discriminant subspace is computed for the first trial (i.e. the first week of data) and then used to compute CE and TI on the next trials. In week 6, an abrupt increase of TI is not accompanied by a trial-to trial change in CE, suggesting a deterministic origin of the observed non-stationary in hourly ozone concentrations. Lowest plot shows the certainty in the classification (see Text S2). (TIF) [file pone.0095648.s002.tif]
