# Supplementary material for: Can We Identify Non-Stationary Dynamics of Trial-to-Trial Variability?
Source: PLoS One. 2014 Apr 25;9(4):e95648. doi: 10.1371/journal.pone.0095648 (PMC4000201; doi:10.1371/journal.pone.0095648)
Supplement: Software S1 — Demo trajectories reconstruction toolbox; pls revise this cite in the text and EM. (ZIP) [file pone.0095648.s003.zip › ntr1_6ReleaseDemo/readMeFirst.pdf]

Neural Trajectories Reconstruction Matlab Toolbox. v1.6b

Current Revision of this text 03/26/2014.

by Emili Balaguer-Ballester. BCCN Heidelberg-Mannheim / Faculty of Science and Technology, Bournemouth University
Contributors:
Daniel Durstewitz, BCCN Heidelberg-Mannheim / Plymouth University
Alejandro Tabas, Faculty of Science and Technology, Bournemouth University
Chris Lapish, Psychology Department, Indiana Purdue University
Jeremy K Semans, Brain Research Centre, University of British Columbia
Marcin Budka, Faculty of Science and Technology, Bournemouth University
Contact: eb-ballester@bournemouth.ac.uk

Updates provided in:

- http://www.bccn-heidelberg-mannheim.de/ [please select "People"/"Durstewitz" or "Balaguer-Ballester" links]
- http://www.researchgate.net/profile/Emili_Balaguer-Ballester/

This is a Beta version, not guaranteed. Nevertheless, support of this "toolbox" for researchers is freely provided by the authors under the terms of the GNU GPL license http://www.gnu.org/licenses/gpl-faq.html#WhatDoesGPLStandFor.

This Matlab© toolbox is based on:

- Balaguer-Ballester E, Tabas-Diaz, A, Budka, M 2014. Can we identify non-stationary dynamics of trial-to-trial variability? PLoS ONE.
- Hyman, J., Ma, L., Balaguer-Ballester, E., Durstewitz, D., Seamans, J.2012. Contextual encoding by ensembles of medial prefrontal cortex neurons. PNAS, 109 (13)5086-5091.
- Balaguer-Ballester E, Lapish C, Seamans JK and Durstewitz D 2011 "Attracting Dynamics of Frontal Cortex Populations during Memory Guided Decision-Making". PLoS Comput. Biol. PLoS Computational Biology, 7(5): e1002057. doi:10.1371/journal.pcbi.1002057.
- Durstewitz, D. and Balaguer-Ballester, E. 2010. Statistical Approaches for Reconstructing Neurocognitive Ensemble Dynamics from High-Dimensional Neural Recordings. Neuroforum, 4 (10): 266-276.

Requirements: Matlab v7.11 or upper (2010b), Signal Processing Toolbox (any compatible version), Statistics Toolbox v7.4 (2010b). It can be easily adapted to work on earlier matlab versions. The code runs either in Windows or in Linux, but please remembers to change the path in the first code lines of "ntr.m" file.

For running a short demonstration of this toolbox please type ">ntr;"

This brief document only provides an overview. Nevertheless, the code is profusely commented. For a detailed help, please move to the deploy directory and type: ">help filename", where "filename" is one of the files located in the main deployment directory. Please, feel free to contact the corresponding author for any question.
****

I. Overview

This toolbox performs statistical analyses on time-series of multivariate neural responses – of different kinds. It is based in the reconstruction of trajectories in suitable expanded spaces. It can be also used in arrange of multivariate time series, please see the setting in which this can be performed in Balaguer-Ballester et al., PLOS ONE.

It is assumed that the separate cognitive epoch's tasks are observable (e.g. a stimulus presentation, a successful choice, reward acquisition, movement to a definite place like an arm) and are labelled accordingly. It is also assumed that all multivariate neural responses are time-ordered and simultaneously recorded. Statistical analyses indicate the probability of those states to behave like attracting regions of responses.

An optimal reconstruction of neural trajectories properties may require high-dimensional spaces. For this reason, kernel methods will be used in the statistical analyses of such state spaces. A demonstrative dataset is provided, based on a rodent performing multiple trials of the task described in Balaguer-Ballester et al (2014, 2011). Please type ">ntr"

II. How-to

II.1) Drop a ".mat" or ASCII-type file (e.g. named 'file_name.mat') on "./data" folder, where "./" indicates the directory containing this entire toolbox aka the "deployment directory" (Note: use ".\" for Linux). This 'file_name.mat' should contain a Matlab© matrix named "data" of dimension "number of time bins x (dimensionality of neural responses+3)", where "dimensionality of neural responses" refers to the number of simultaneously recorded neurons, variables, voxels, electrodes etc. and/or delayed version of them. The dataset structure is the following:

- Columns "1:end-3" of "Data" matrix: Must contain neural responses over time.
- Column "end-2" of "Data" matrix: Must contain natural numbers>0, labelling the different stimuli or behavioural "epochs" in which the experimentalist segments the task. "-1" should encode "no-labelled" time-bins.
- Column "end-1" of "Data" matrix: Must contain natural numbers>0, they are alternative labelling used only for trial trajectory display (number of labels has to be smaller than 8. See comments in file for more info). Those labels typically represent "phases" of the task, containing different "epochs". If all "epochs" are to be displayed, this column has to be a copy of "end-2" one.
- Last column of "Data" matrix: Must contain natural numbers>0, labelling the different trials of the task. Trials typically represents repetitions of the experiment, containing each the same "phases" of the task.

II.2) Open "kspaces_configuration.m" and setup the configuration parameters. Please find a detailed description of the parameters by typing ">help kspaces_config".
II.3) Once in the deployed directory, type ">ntr('file_name');". Alternatively, one can load in the workspace the data matrix variable formatted as indicated in I.1 (e.g. "Data_matrix_name") and type ">ntr(Data_matrix_name);".

III. Parameters

Parameters are specified in "kspaces_config.m" file. They are subdivided (for clarity purposes) into "Basic" and "Advanced" parameters. In this brief note, only few parameters are described. Please type ">help kspaces_config" and read "setup_config.m" function (and on each file of this toolbox) for more information.

III.1) Configuration of the algorithm is achieved by the next parameters:

- "order": Indicates the maximum order of the polynomial products among units' responses (please see "multinom_kernel.m" for more information). This order is the parameter defining each high-dimensional space.
- "regularization": Ensures the validity of the analyses in future dataset by "penalizing" the complexity of the Fisher Discriminant Criterion in high-dimensional spaces. It has to be tuned by cross-validation analyses for each particular kind of data (please see "kfd_multiv.m"; "class_trajec.m" and "kfd_cross_val.m" for more information).
- "is_shuffled_events": If positive, shuffles blocks as specified by the labels in "end-2" data column (task-epoch labels). Please see "shuff_data.m" for more information. This serves for creating non-parametric block-permutation bootstrap analyses in which short-term autocorrelation is typically preserved.
- "is_shuffled_within_events": If positive, preserves blocks as specified by the labels in "end-2" data column (task-epoch labels), but shuffles all time-bins within such task-epochs (please see "shuff_data.m" for more information). This serves for creating non-parametric bootstrap analyses in which temporal contingency is destroyed.
- "do_dcm": If positive, performs a delay-coordinate map for disambiguating trajectories i.e. dimensions constructed from delayed versions of units' responses will be added to the original multivariate recordings space. An unwanted trajectory crossing occurs when all neural responses dimensions corresponding to two or more district task-epochs have the same values in the same time bin. Delay-coordinate maps may typically avoid those ambiguities. Other advanced parameters control how this map will be performed (see "dcm.m" for more information).

III.2) Low-dimensional displays:

- Single-trial trajectory display. Only shown if the configuration field "trial_disp>0" or "make_video>0". Displays a single-trial trajectory for orders 1 and the selected one for visual comparison (left and right plots respectively), coloured according to the task-phases selected in the Column "end-1" of "Data" matrix (see II.1). This plot is obtained using *kernelized* principal components analyses (K-PCA, Schölkopf et al, 1998) of a delay-coordinate map. The display includes only one trial, the one selected in the configuration field "trial_disp" (see "kspaces_config.m"). Alternatively, if the configuration field "make_video>0", a video of such trial will be played instead. More information by typing ">help visualization" (see also "visualization.m")
- Multiple trials flow display. Only shown if the configuration field "trials_flow_disp>0". Displays flow field for orders 1 and the selected one for visual comparison (left and right plots respectively), coloured according to the task-epochs selected in column

"end-2" of "Data" matrix (see II). Those were plots into the three main first discriminant axes (or two if there are less than three task-epochs), obtained by multivariate *kernel*-fisher discriminant analyses (K-FDA, Mika et al., 2000). The display includes several consecutive trials simultaneously, the lower and upper and ones appear in the 1x2 matrix "trials_flow_disp" (see "kspaces_configuration.m").

III.3) Command window statistical reports, showing out-of-sample predictions (cross-validation) in high-dimensional spaces (defined by the multiple activity interactions; see previous paragraphs) of task-epochs vs. neural states associations. Two types of statistics can be found: classical (comparing percentage of miss-classified vectors for each task-epoch and across different validation sets, see below) and a trajectory-based analysis (comparing percentage of divergent trajectories for each behavioural epoch).

- Multiple-discriminant analyses. A Two-class FDA i.e. a maximum discriminating solution corresponding will be used for trajectory analyses. In addition, if "is_multiple_discriminant>0", an optimal discriminating subspace of dimensions = number of task-epochs-1 will be generated i.e. all task-epochs will be simultaneously analysed (default).
- Full statistics report. Default displays do not show statistical tests, intermediate statistical reports and warnings related to numerical problems in the analyses. However, iIf "full_stats_disp>0", information-theoretic measures, parametric and non-parametric normality tests of different kind and all warnings and intermediate reports will be displayed (please see "class_trajec.m" and "kfd_multiv.m" for more details). Warning: command window may become too "crowded", not recommended as default approach.
- Cross-validation. Different kinds of out-of-sample predictions are supported. If "causal_xval<0", standard leave-one-out method will be performed: Each ith-trial is removed in turn, then optimum discriminant directions are computed using all-but-the ith trial; while this trial will become the ith-validation set. However, if "causal_xval>0", causality-preserved n-fold cross-validation will be performed instead: last j-trials are removed in each j-th validation block. The non-removed trials form the reference set (which thus is smaller for increasing j-th-validation blocks). There will be n=(m/2)-1 validation blocks, always occurring "after" the reference set. Please see comments in the file for more information and other validation modalities.
