# Supplementary material for: Can We Identify Non-Stationary Dynamics of Trial-to-Trial Variability?
Source: PLoS One. 2014 Apr 25;9(4):e95648. doi: 10.1371/journal.pone.0095648 (PMC4000201; doi:10.1371/journal.pone.0095648)
Supplement: Text S1 — Local trajectory analyses in a Duffing system. (PDF) [file pone.0095648.s004.pdf]

Text S1. Local trajectory analyses in a Duffing system

This supplemental section further analyses the behaviour of trajectories in the Duffing system. Figures 2 and 3 in the main text show examples of discrete trajectories switching their basins of attraction i.e. their class when the parameter α changes in Equation (1). Here we will quantitatively show how for a given parameter perturbation there is a trajectory, transiting close to the origin, which potentially changes its basin of attraction.

Consider the Duffing system,

$$\ddot{x}(t) + \delta\dot{x}(t) - \beta x(t) + \alpha x^3(t) = \Omega \cdot \cos(wt) \tag{1}$$

exhibiting a dynamical landscape consisting of two attracting spirals and a centre (see parameter values in Results section and e.g. [1,2]). Despite its simplicity there are no parsimonious analytical solutions for such model in the general case (to our knowledge, see e.g. [2–5]). For instance, Feng and colleagues [2] recently found an integral of motion in terms of Jacobi elliptic functions [6], which does not correspond to the bi-stable dynamics used in our work. Thus, we base our analyses in locally studying the behaviour of the system close to the fixed points.

First, we show how, for an arbitrary trajectory transiting in the neighbourhood of one of the attractors, a parameter change $\Delta\alpha > 0$ yields to a change in the phase space ordinate $\delta\dot{x}$. We focus on the vicinity of the positive attractor $x_0 = \sqrt{\frac{\beta}{\alpha}}$, where:

$$x^3 \simeq x_0^3 + 3x_0^2(x - x_0) + \mathcal{O}\left((x - x_0)^2\right) \tag{2}$$

After simple algebra, Equation (1) can be locally linearized,

$$\ddot{x} + \delta\dot{x} + 2\beta(x - x_0) = 0 \tag{3}$$

which has the trivial solution [1]

$$x(t) = e^{-\frac{\delta}{2}t}\left(ae^{wt} + be^{-wt}\right) + x_0 \tag{4}$$

where $w = +\sqrt{\left(\frac{\delta}{2}\right)^2 - 2\beta} \in \mathbb{C}$, and thus the phase space ordinate is

$$\dot{x}(t) = -\frac{\delta}{2}x(t) + we^{-\frac{\delta}{2}t}\left(ae^{wt} - be^{-wt}\right) \tag{5}$$

Note that the dependency on α in $x(t)$ is the attractor position x_0.

Now we will consider a trajectory $(\hat{x}, \dot{\hat{x}})$ with the same initial conditions $(x(t_0), \dot{x}(t_0))$ but in a system where the perturbation term has changed (the perturbed system). When α increases, the attractor $x_0 \longrightarrow \hat{x}_0$ approaches the origin by $\varepsilon = \sqrt{\frac{\beta}{\alpha+\Delta\alpha}}$. Thus, the initial conditions must satisfy:

$$x(t_0) = \hat{x}(t_0) = x_0 + \varepsilon \tag{6}$$

$$\dot{x}(t_0) = \dot{\hat{x}}(t_0) = \kappa \tag{7}$$

where $\kappa < 0$ is the initial condition $\dot{x}(0)$ i.e. we have chosen a trajectory in the $x > 0$, $y < 0$ quadrant and thus approaching the centre $x = 0$. For small $\Delta\alpha$ and after simple algebra,

$$\hat{x}_0 \equiv \sqrt{\frac{\beta}{\hat{\alpha}}} = \sqrt{\frac{\beta}{\alpha}}\left(1 - \frac{\Delta\alpha}{2\alpha}\right) + \mathcal{O}\left(\left(\frac{\Delta\alpha}{\alpha}\right)^2\right) \simeq x_0\left(1 - \frac{\Delta\alpha}{2\alpha}\right) \tag{8}$$

which for $t_0 = 0$ (without loss of generalization) implies

$$a = \frac{1}{2}\left(\varepsilon + \frac{\kappa}{\omega} + \frac{\delta}{2\omega}\varepsilon\right) \tag{9}$$

$$b = \frac{1}{2}\left(\varepsilon - \frac{\kappa}{\omega} - \frac{\delta}{2\omega}\varepsilon\right) \tag{10}$$

$$\hat{a} = a + \frac{\Delta\alpha}{4\alpha} \tag{11}$$

$$\hat{b} = b + \frac{\Delta\alpha}{4\alpha} \tag{12}$$

where $\hat{a}, \hat{b}$ are the constants of the exact solution to the distorted trajectory (5).

Thus at $t_0+\Delta t$, using Equations (4), (11) and (12), the new position of the trajectory in the perturbed system $\hat{x}(t_0+\Delta t)$ is

$$\hat{x}(t_0+\Delta t) - x(t_0+\Delta t) = \frac{\Delta\alpha}{4\alpha} e^{\frac{-\delta}{2}(\Delta t+t_0)}\left(e^{w(\Delta t+t_0)} + e^{-w(\Delta t+t_0)}\right) \tag{13}$$

which, for small Δt and $t_0 = 0$, can be easily approximated to

$$\hat{x}(t_0+\Delta t) - x(t_0+\Delta t) \simeq \frac{\Delta\alpha}{2\alpha} \tag{14}$$

Thus $\hat{x}(t_0+\Delta t) > x(t_0+\Delta t)$ i.e. $\dot{\hat{x}}(t_0+\Delta t) < \dot{x}(t_0+\Delta t)$ and thus trajectory behaviour in the proximity of the attractor differ for the perturbed system (the same results applies to the left attractor due to the symmetry of the system).

Next we will show that any two trajectories of this kind i.e. approaching the origin from different phase space locations potentially converge to different attractors.

Lemma 1. *Consider the autonomous Duffing System* (1) *in the dynamical regime described above. For any ε there always exist two trajectories $x_1(t)$, $x_2(t)$ satisfying the following two sets of conditions in a vicinity of the origin:*

1. *At $t = t_0$ both trajectories approach the origin from $x > 0$ form different vertical positions in the phase space:*
   - *(a)* $x_1(t_0) = x_2(t_0) = \theta, \quad \theta \approx 0 \ll \beta/\alpha$
   - *(b)* $\dot{x}_1(t_0), \dot{x}_2(t_0) < 0$
   - *(c)* $\dot{x}_2(t_0) - \dot{x}_1(t_0) = \varepsilon$
2. *At ($t_0 + \Delta t \approx 0$) both trajectories are located in different quadrants of the phase space:*
   - *(a)* $x_1(t_0+\Delta t) = -x_2(t_0+\Delta t)$
   - *(b)* $\dot{x}_1(t_0+\Delta t) = -\dot{x}_2(t_0+\Delta t)$

Proof. In a neighbourhood of the origin and at $x_0 = 0$, the Duffing System (1) can be easily linearized. Thus, for $\|x\| \ll \beta/\alpha$ and using Equation (2), the system is approximated by:

$$\ddot{x} + \delta\dot{x} - \beta x \approx 0 \tag{15}$$

which has a trivial solution

$$x(t) = e^{-\frac{\delta}{2}t}\left(ae^{\omega t} + be^{-\omega t}\right) \tag{16}$$

where $\omega = \sqrt{\left(\frac{\delta}{2}\right)^2 + \beta}$ and $\omega \in \mathbb{R}$, $\omega > \frac{\delta}{2}$; note that this solution is independent from the perturbation term α. The derivative of (16) is given by (5); here the coefficients a and b are named a_1, b_1 and a_2, b_2 for trajectories $x_1(t)$ and $x_2(t)$, respectively.

Condition 1a.At $t_0 = 0$ (without loss of generality) the condition 1a can be expressed as

$$a_i + b_i = \theta \qquad i = 1, 2 \tag{17}$$

Conditions 1b and 1c Rewriting the conditions as $\dot{x}_1(t_0) = \kappa$ and $\dot{x}_2(t_0) = \kappa + \varepsilon$ (with $\kappa + \varepsilon < 0$) and using Equation (5) they are equivalent to

$$\eta a_1 - \mu b_1 = \kappa \tag{18}$$

$$\eta a_2 - \mu b_2 = \kappa + \varepsilon \tag{19}$$

with $\eta = \omega - \delta/2 > 0$ and $\mu = \omega + \delta/2 > \eta$. After simple algebra, Equations (17), (18) and (19) imply the next equalities:

$$a_1 = \frac{1}{\mu + \eta}(\mu\theta + \kappa) \tag{20}$$

$$b_1 = \frac{1}{\mu + \eta}(\mu\theta - \kappa) \tag{21}$$

$$a_2 = a_1 + \frac{\varepsilon}{\mu + \eta} \tag{22}$$

$$b_2 = b_1 - \frac{\varepsilon}{\mu + \eta} \tag{23}$$

$$\tag{24}$$

This system has solutions for any ε and $\theta > 0$ if and only if

$$b_1 > 0, \quad a_2 > a_1 \quad \text{and} \quad b_2 < b_1 \tag{25}$$

which completes the proof.

Conditions 2a, 2b Using Equation (16) at $t_0 = 0$,

$$(a_1 + a_2)e^{\omega \Delta t} + (b_1 + b_2)e^{-\omega \Delta t} = 0 \tag{26}$$

$$(a_1 + a_2)e^{\omega \Delta t} - (b_1 + b_2)e^{-\omega \Delta t} = 0 \tag{27}$$

Respectively. As exponentials are definite positive, Equations (26) and (27) are uniquely satisfied if $a_1 = -a_2$ and $b_1 = -b_2$; those values are compatible with restrictions shown in Equation (25) just by choosing $a_1 < 0$, which completes the proof.

□

Using this lemma we can derived the next proposition:

Proposition 1. *Given the unforced Duffing System described above and a perturbation $\Delta\alpha$, at least one of the trajectories changes its basin of attraction. The inverse is also true i.e. if a trajectory changes its basin of attraction there is a perturbation value $\Delta\alpha$ which can account for this effect.*

An intuitive demonstration is the following: Consider two arbitrary trajectories $x(t)$ and $\hat{x}(t)$ of two Duffing systems in the dynamic regime described above; where the perturbation term is α in the former and $\hat{\alpha} = \alpha + \Delta\alpha$ in the later. The two trajectories have the same initial conditions in the vicinity of the right attractor $x_0 = \pm\sqrt{\frac{\beta}{\alpha}}$.

From Equation (14) these two trajectories will depart shortly thereafter. Due to the causality of the phase space, trajectories will approach the centre $x = 0$ at different horizontal speed i.e. their vertical coordinate in the phase space will differ in the vicinity of the origin.

Therefore, using the Lemma 1, after a small Δt the two trajectories will be located in diagonally opposite quadrants of the space (Equations (2a), (2b)) and at the same Euclidean distance from the origin i.e. closer to different attractors and moving in different directions by the same amount. Thus, by symmetry of the system (see for instance Figure 2B) the two trajectories belong to different basins of attraction.

Conversely, two trajectories belonging to different basin of attraction approach the origin differently according to 1 and thus can be related to a parameter change (Equation (14)).

References

1. Wiggins S (2013) Introduction to applied nonlinear dynamical systems and chaos. Springer.

2. Feng Z, Chen G, Hsu S (2006) A qualitative study of the damped duffing equation and applications. American Institute of Mathematical Sciences 6: 1097 – 1112.

3. Holmes P, Whitley D (1983) On the attracting set for duffing's equation. Phys D 7: 111-123.

4. Du J, Cui M (2010) Solving the forced duffing equation with integral boundary conditions in the reproducing kernel space. International Journal of Computer Mathematics 87: 2088-2100.

5. Sabarathinama S, et al (2013) Transient chaos in two coupled, dissipatively perturbed hamiltonian duffing oscillators. Communications in Nonlinear Science and Numerical Simulation 18: 3098–3107.

6. Gradstein IS, Ryzhik IM (2007) Tables of Integrals, Sums, and Products. Elsevier.
