# Supplementary material for: Can We Identify Non-Stationary Dynamics of Trial-to-Trial Variability?
Source: PLoS One. 2014 Apr 25;9(4):e95648. doi: 10.1371/journal.pone.0095648 (PMC4000201; doi:10.1371/journal.pone.0095648)
Supplement: Text S2 — Illustrative dataset in a non-stationary environment. (PDF) [file pone.0095648.s005.pdf]

Text S2. Illustrative dataset in a non-stationary environment

This supplementary gives an illustration of the analytical results obtained in a well-known oscillatory multivariate time series where attractors subtly drift over time. Figure S2A shows the hourly ozone (O_3) ground concentration, nitric oxides (NO_2, NO) temperature and relative humidity over the period of a week. Ozone is an atmospheric pollutant synthesised from a series of precursor gases (mainly NO_2) by the catalysis of solar radiation.O_3 levels were grouped in three classes (low, moderate and high), and a discriminant defined in an optimally expanded phase space was then used to map precursors and atmospheric variables (Figure S2A, bottom) to the three O_3 ranges.

Given the complexity of the atmospheric system dynamics, it is very difficult to discern the deterministic processes involved in the seasonal, non-stationarity trend observed in O_3 concentrations. We will address this issue empirically in a simple example with the analyses developed here.

Dataset. Elevated concentrations of tropospheric ozone are known to affect human health and vegetation adversely, thus time series of O_3 concentrations have been extensively studied for decades e.g. [1-3]. Ozone concentration is known to exhibit regular daily oscillations (Figure S2A) and seasonal behaviour; with maximum concentrations and stronger periodicity in summer, becoming progressively weaker during the autumn weeks. Thus, this dataset is particularly useful as an illustration of our analyses. Data used in this research belongs to the Department of Agriculture, Generalitat Valenciana (Regional Government), Valencia, Spain; and it was recorded in a rural area of particular agricultural interest (39º 7’ N; 0º 27’ W). The station has continuously monitored ambient concentrations of pollutants (Dasubi 1008 RS, 2108, 8001) as well as meteorological variables mentioned above (Skye SKH 2013/1) since 1996 (8 weeks from August 12 were used here). Data was collected and averaged over 60 min intervals (for more details in data pre-processing, see [2])

Analyses. The original phase space was spanned by the hourly averaged concentrations of NO_2, NO and hourly mean values temperature and relative humidity. Data was centred and normalized and no missing values were reported during the period used. Optimally regularized kernel discriminant operating on a third order expanded space (see methods) was used to map the ozone ranges to the phase space variables. Regularization penalty (here the optimal penalization constant was 4.8% of the mean kernel matrix, see [4] for details on the process) of the discriminant was optimized by cross validation on TD on a separate dataset. Classification subspace was then computed for the first trial (a week of hourly recordings) and held fixed for the following trials.

Results are shown in Figure S2B. Classification error and trajectory divergence increase monotonically until week 8, where TD increases abruptly and CE does not. This change occurs precisely at trial 8, when CE exceeds the confidence level at trial 1. To benchmark further the results, bootstraps (n=200) were constructed by shuffling the temporal order within trajectories (yet not shuffling across trials). As expected, bootstrap data do not show any trend. Figure S2B (inset) also shows an alternative measure to CE, namely the certainty of the classification which in our case is simply the normalized difference between the highest and the lowest discriminate z-score of all classes [5]. Interestingly, the increase in TD is indicated in this system by a sharp decrease in the average certainty.

In summary, as in Duffing dynamical system simulations, this result suggests a deterministic trend underlying the observed variability of ozone concentrations as would be expected from previous studies (e.g. [1-3]).

References

1. Gómez-Sanchis J, Martín-Guerrero, JD, Soria-Olivas, E, Vila-Francés J, Carrasco, JL, del Valle-Tascón, S (2006) Neural networks for analysing the relevance of input variables in the prediction of tropospheric ozone concentration. Atmospheric Environment, 40 (32): 6173-6180.

2. Pastor-Bárcenas O, Soria-Olivas E, Martín-Guerrero JD, Camps-Valls G, Carrasco-Rodríguez JL, del Valle-Tascón S (2005) Unbiased sensitivity analysis and pruning techniques in neural networks for surface ozone modelling. Ecological Modelling, 182 (2): 149-158.

3. Balaguer-Ballester E, Camps-Valls G, Carrasco-Rodriguez JL, Soria E, del Valle-Tascon S (2002) Effective one-day ahead prediction of hourly surface ozone concentrations in eastern Spain using linear models and neural networks, Ecological Modelling, 156: 27-41.

4. Balaguer-Ballester E, Lapish C, Seamans J, Durstewitz D (2011) Attracting dynamics of frontal cortex ensembles during memory-guided decision making. PLoS Computational Biology, 7(5): e1002057.

5. Schapire RE, Freund Y, Bartlett P, Sun Lee W (1998) Boosting the margin: A new explanation for the effectiveness of voting methods. The annals of statistics 26(5): 1651-1686.
